# Heart rate and heart rate variability of dairy cows with or without local anesthesia

**DOI:** 10.3168/jdsc.2025-0803

**Published:** 2025-12-04

**Authors:** Zs. Bohák, H. Kiszlinger Nagyné, F. Hoffmann, L. Kern, L. Kovács

**Affiliations:** 1 Department of Animal Husbandry and Animal Welfare, Institute of Animal Sciences, Hungarian University of Agriculture and Life Sciences, Gödöllő, H-2100 Hungary; 2 Bona Adventure Ltd., Gödöllő, H-2100 Hungary

## Abstract

Heart rate variability analysis provides valuable insights into autonomic nervous system function but remains minimally explored in veterinary anesthesiology, particularly regarding its relationship with low-dose caudal epidural anesthesia in cattle. This study employed a crossover design to determine whether epidural lidocaine administration (4 mL of 2% solution) affects cardiac autonomic regulation compared with physiological saline in 8 healthy Holstein-Friesian dairy cows. Interbeat intervals were measured at baseline and for 240 min following epidural injection and cardiac autonomic activity-related parameters of heart rate variability (heart rate, root mean square of successive differences, high-frequency component, and ratio of low-frequency and high-frequency components) were calculated. Area under the curve for the first 240 min after the treatment and time to return to baseline were used to evaluate overall treatment effects on cardiac autonomic activity. Results revealed no differences in the area under the curve values for any parameters of heart rate variability between lidocaine and saline epidural interventions throughout the observation period, with comparable recovery times to baseline values between treatments. These findings suggest that sacrococcygeal epidural anesthesia did not detectably affect cardiac autonomic regulation in dairy cows under the conditions of this study.

## Highlights

• Caudal epidural lidocaine does not affect HRV in dairy cows.
• HRV remains stable during epidural anesthesia, validating welfare studies in cattle.
• The crossover design confirmed no autonomic effects from sacrococcygeal injection in cattle.
• The low-dose technique preserves cardiac autonomic regulation in cattle.

## Graphical Abstract

**Figure:**
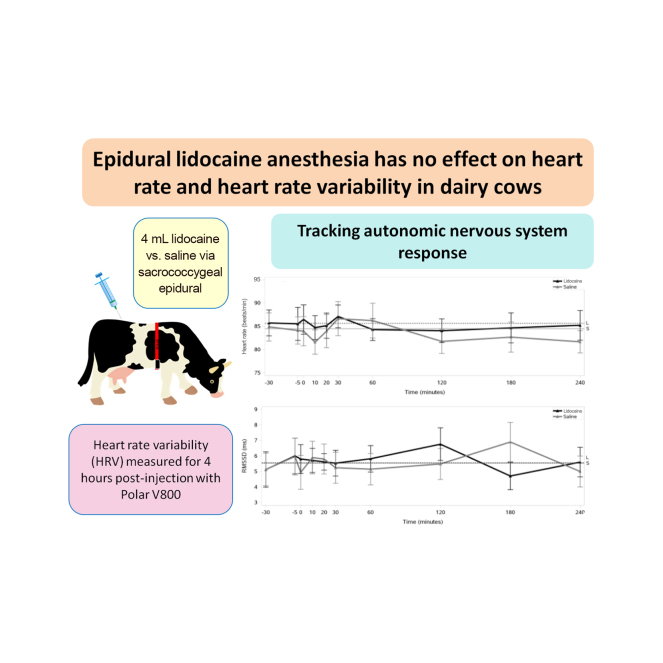
**Summary:** This study investigated whether low-dose caudal epidural lidocaine administration affects cardiac autonomic regulation in dairy cows, a question crucial for validating heart rate variability (HRV) as a welfare assessment tool. Using a crossover design, 8 Holstein-Friesian cows received either 4 mL of 2% lidocaine or physiological saline via sacrococcygeal epidural injection. Heart rate and HRV parameters (root mean square of successive differences [RMSSD], high-frequency [HF], ratio of low-frequency and high-frequency components [LF/HF]) were monitored for 240 minutes postinjection using Polar V800 Polar Electro OY, Kempele, Finland) devices. Results showed no significant differences in area under the curve values for any HRV parameter between lidocaine and saline treatments. These findings demonstrate that sacrococcygeal epidural anesthesia does not detectably alter cardiac autonomic function in dairy cows. This validation supports the use of HRV analysis in welfare studies involving epidural anesthesia, confirming that observed HRV changes during procedures can be attributed to stress responses rather than anesthetic effects.

Farm animals present unique challenges in anesthetic management. The risk of regurgitation and ingesta inhalation after general anesthesia is substantially higher in these animals compared with other domestic species, which has led veterinarians to prefer physical restraint combined with local or regional analgesia techniques (Fierheller et al., 2004; Lee and Yamada, 2005). Caudal epidural analgesia using local anesthetics has been an established and widely used technique in cattle for several decades (Elmore, 1980; Skarda and Muir, 1979; Skarda, 1986). Lidocaine hydrochloride is the most commonly used local anesthetic drug for epidural analgesia in cattle with a dose between 0.11 and 0.22 mg/kg of 2% solution (Saifzadeh et al., 2007; Azari et al., 2014; Atiba et al., 2015).

Heart rate variability (**HRV**) measures variations in time between consecutive heartbeats, providing insights into autonomic nervous system (**ANS**) functioning (von Borell et al., 2007). In anesthesiology research, HRV analysis is used to assess ANS responses to anesthetic interventions and evaluate the effectiveness of regional anesthetic techniques in maintaining autonomic balance. Although most research has focused on humans (Fleisher et al., 1994; Deschamps et al., 2004; Owczuk et al., 2009; Song et al., 2017; Wujtewicz and Owczuk, 2023), these principles show promise for veterinary applications, particularly for understanding epidural anesthesia effects on ANS function.

Human studies using different epidural techniques (lumbar vs. thoracic injection sites, various local anesthetics and concentrations) show varied ANS effects on HRV (Hopf et al.,1995; Fleisher et al., 1994; Deschamps et al., 2004; Song et al., 2017). In veterinary research, this relationship has been minimally explored. Despite numerous studies on epidural anesthesia in cows (Skarda, 1996; Grubb et al., 2002; Al-lethie et al., 2023), no research assessed the cardiac autonomic responses to the treatments.

Animal welfare studies increasingly rely on HRV analysis, yet researchers face uncertainty about whether epidural anesthesia itself influences HRV parameters independently of the procedural stress being measured. It should be ruled out that lidocaine absorption affect cardiac function. For example, when assessing animal welfare during reproductive procedures such as artificial insemination or embryo transfer where caudal epidural anesthesia is routinely used, researchers need to distinguish between stress-induced HRV changes and anesthesia-related autonomic effects. This differentiation is crucial for accurate welfare assessment and for developing more humane handling protocols. This study aimed to determine whether epidural lidocaine administration directly affects cardiac rhythm in cattle or if HRV changes occur independently of the injected substance (lidocaine vs. physiological saline).

The experiment was carried out between October 9 and 10, 2024, on a large-scale dairy farm in Hungary with a herd of 900 lactating cows. The research protocol employed a crossover design involving 8 healthy second-parity Holstein-Friesian cows with (means ± SD) BCS 2.7 ± 0.1; age 4.3 ± 0.3 yr, DIM 135.8 ± 9.2, milk yield 42.1 ± 3.0 kg/d, and BW ranging from 480 to 520 kg (median: 498 kg, mean ± SD: 501 ± 12 kg). This resulted in a lidocaine dose range of 0.15 to 0.17 mg/kg (mean: 0.16 ± 0.01 mg/kg), which falls within the recommended dose range of 0.11 to 0.22 mg/kg for epidural anesthesia in cattle (Saifzadeh et al., 2007; Azari et al., 2014; Atiba et al., 2015). The selected animals were confirmed to be nonpregnant during the experimental period. Cows were housed in modern freestall barns bedded with sand in groups of ∼70 animals per pen. The 8 experimental animals were selected from and remained within this social group throughout the study period. A TMR was fed once a day at 0800 h, and animals had free access to water. Cows were milked 3 times a day in a 2 × 28-stall parallel Bosmark milking parlor (Bosmark Kft., Biatorbágy, Hungary).

Animals were randomly allocated to treatment sequence using a computer-generated randomization list (random number generator in Excel, Microsoft Corporation). On the first experimental day, 4 randomly selected animals received 4 mL of 2% lidocaine (**LID**; Lidobel inj. 20 mg/mL, Bela-Pharm GmbH & Co., Germany) via epidural injection, whereas the remaining 4 animals were administered an equivalent volume of physiological saline (**SAL**; Salsol A, Teva Pharmaceutical Works Ltd., Debrecen, Hungary). After a 1-wk washout period, the experiment was repeated with reversed group assignments (crossover design), with animals that previously received lidocaine now receiving saline, and vice versa.

Before the measurement of the interbeat intervals (**IBI**), animals were restrained exclusively within their customary barn freestalls to reduce stress related to measurement preparation. One hour subsequent to feeding, electrode belts were positioned on the cows as advised by Kovács et al. (2014), and to optimize conductivity, electrode sites were covered with ultrasound transmission gel. Bluetooth signals from the Polar H10 sensor (Polar Electro OY, Kempele, Finland) were captured using a Polar V800 watch (Polar Electro OY, Kempele, Finland; Wierig et al., 2018), which was securely attached to the animals' activity collar. The cows were permitted to wear the monitoring equipment unrestricted for one hour, facilitating device acclimatization.

The animals were subsequently returned to the freestalls and received an epidural injection, adhering to the methodological approach previously described by Al-lethie et al. (2023). Low-dose caudal epidural anesthesia was performed using 4 mL of 2% lidocaine, which preserves motor function of the hind limbs and limits anesthetic effects to the sacral and coccygeal segments without affecting lumbar nerve fibers. The sacrococcygeal region underwent aseptic preparation. Animals were minimally restrained in freestalls using a simple rope barrier to prevent backward movement during injection. With the animal maintaining a standing position, the epidural injection was administered through the sacrococcygeal space utilizing a sterile 18-gauge 3.7-cm long needle, with the bevel oriented forward. Accurate needle placement was verified through the characteristic loss of resistance during drug injection.

After the epidural injection, the animals remained stationary in the freestall for 5 min to ensure the physiological effects of the lidocaine. Analgesia onset was systematically evaluated by applying a standardized needle pin prick stimulus across multiple anatomical regions, including the tail, anus, perineum, vulva, and inguinal area (Marzok and El-khodery, 2016). Analgesia was operationally defined as the complete absence of movement in response to pin pricks (Al-lethie et al., 2023). For LID cows, sensory loss occurred consistently within a 5-min window. Following analgesia confirmation, the animals were immediately released back into their home freestall barn, where they were housed together with their 70 pen-mates in their familiar social group. This approach ensured that animals maintained their normal social environment and routine behaviors during the 4-h monitoring period, minimizing potential confounding effects of social stress, separation anxiety, or environmental changes on HRV measurements. Throughout the 4-h post-epidural intervention observation period, cows were monitored for any potential physiological or behavioral anomalies, including signs of ataxia, overknuckling, hind limb weakness, or other motor deficits. No abnormal behavioral manifestations were detected.

Interbeat intervals were analyzed for equal 5-min time intervals; 2 for baseline (at 30 and 5 min preceding epidural injection), and at 0, 10, 20, 30, 60, 120, 180, and 240 min after the treatment. Analysis of the IBI was performed using the Kubios HRV software (version 4.1.2.1). Artifact correction employed a custom filtering algorithm that identified IBI deviating from the preceding IBI by more than 30% as artifacts. To eliminate slow nonstationary trend components, the “smoothness priors”-based detrending method was implemented, with λ calibrated to 1,000 and cutoff frequency (fc) to 0.029 Hz.

Time domain measurements included heart rate (**HR**) and the root mean square of successive differences (**RMSSD**) which reflects vagal tone in dairy cattle (Kovács et al., 2014). Frequency-domain HRV analysis was conducted through fast Fourier transformation. The HRV spectrum was calculated via Welch's periodogram method using 256-s overlapping segments with a 50% window overlap. The IBI series interpolation rate was standardized to 4 Hz. Spectral parameters included the normalized power of high-frequency (**HF**) component and the ratio of the low-frequency (**LF**) and HF components (**LF/HF**). The HF component reflects the vagal modulation of the heart, whereas the LF/HF provides information on the sympathovagal balance in dairy cattle (Kovács et al., 2014). Spectral limits for LF and HF were set at 0.05 to 0.20 Hz and at 0.20 to 0.58 Hz, respectively (von Borell et al., 2007).

Statistical analyses were performed using SAS software. The HRV values of the SAL and LID groups at each time point were compared using a paired *t*-test or a Wilcoxon signed-rank test, depending on the results of the normality test. Baseline HRV parameters were calculated by averaging measurements taken at −30 and −5 min before epidural administration and were compared between treatments using the Fisher's exact test.

To evaluate the overall effect of treatments on HRV parameters throughout the measurement period, area under the curve (**AUC**) for the response parameter (AUC_RESP_) was calculated for each parameter using the trapezoidal method described by Lay et al. (1996) as follows:

AUC_RESP_ = Σ[(P_n_ + P_n+1_)/2 × m – BASELINE],

where P is the response parameter at a given time point, m is the time in minutes between the 2 P values, and BASELINE is the baseline value for the given HRV parameter. The AUC values between LID and SAL treatments were compared using a paired *t*-test for normally distributed data or by the Wilcoxon signed-rank test for data showing nonparametric distributions. For all statistical analyses, *P* < 0.05 was considered significant.

The temporal changes in HRV parameters over the 240-min observation period are shown in Figure 1, Figure 2. Both treatments showed similar patterns of HR fluctuation, with values generally remaining within 80 to 87 beats/min (Figure 1a), and no differences were found over time within each treatment. Similarly, RMSSD demonstrated comparable variability between treatments **(**Figure 1b). The HF showed some variation over time in both treatments, but no differences were found between time points within either treatment group (Figure 2a). Similar patterns of fluctuation over time were observed for LF/HF in both treatments without any difference between the time points of measurement.

**Figure 1:**
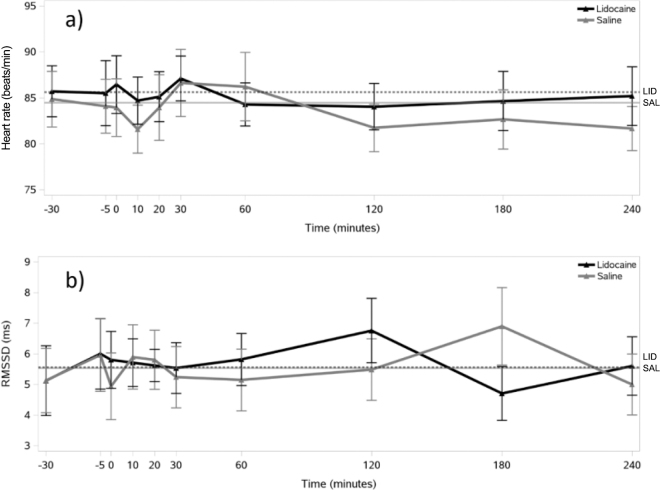
Temporal changes in (a) heart rate (HR) and (b) the root mean square of successive differences in the consecutive IBI (RMSSD) relative to epidural administration of lidocaine (n = 8) or saline (n = 8) in lactating Holstein-Friesian cows, plotted with error bars representing the SEM at each time point. The injection was administered to cows at 0 min. Baseline measurements were taken at −30 and −5 min before the injection. Horizontal lines represent baseline values (solid line = saline group; dotted line = lidocaine group).

**Figure 2:**
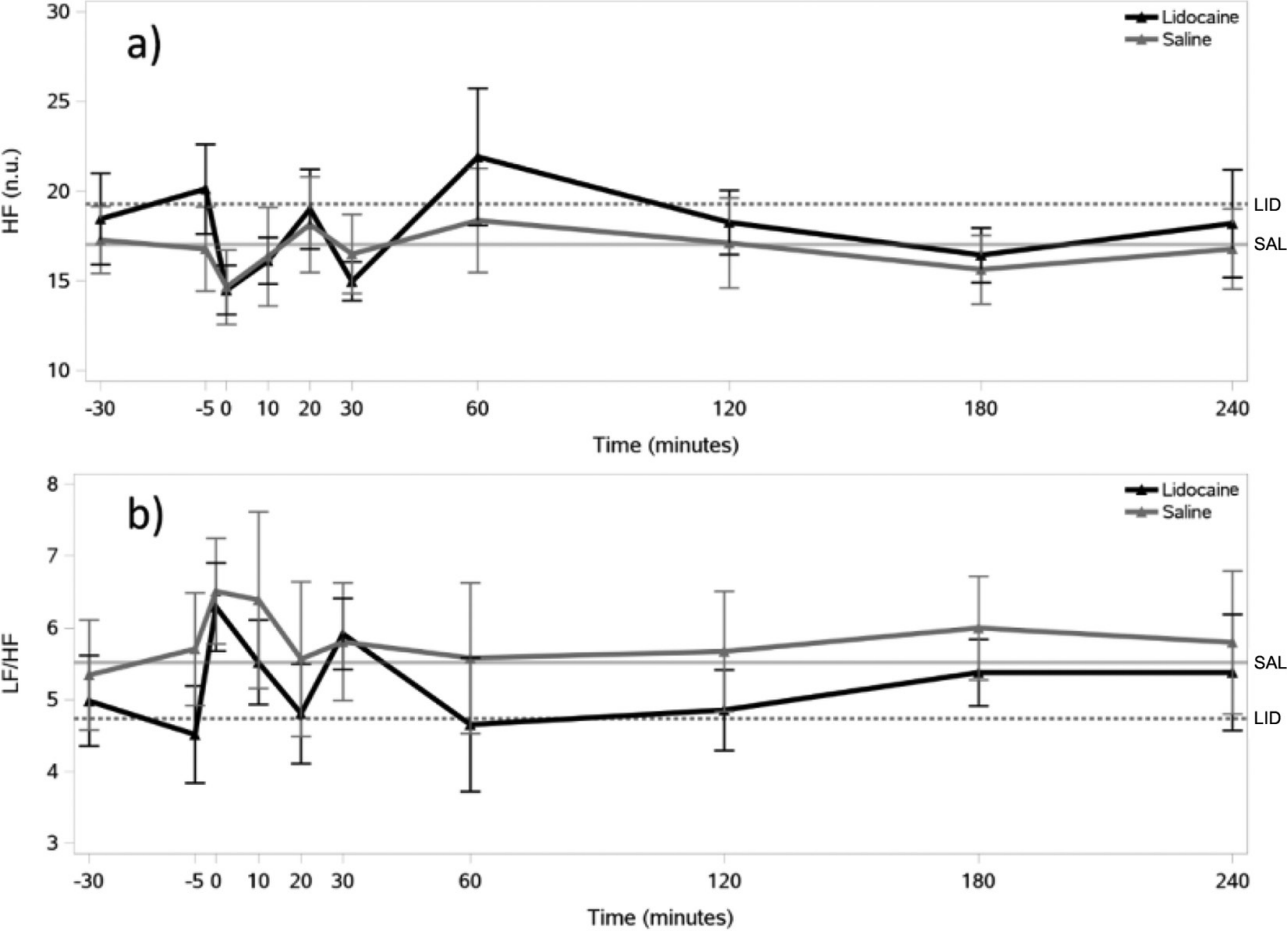
Temporal changes in (a) the high-frequency power of heart rate variability (HF) and (b) the ratio between the low-frequency (LF) and HF components (LF/HF) relative to epidural administration of lidocaine (n = 8) or saline (n = 8) in lactating Holstein-Friesian cows, plotted with error bars representing the SEM at each time point. The injection was administered to cows at 0 min. Baseline measurements were taken at −30 and −5 min before the injection. Horizontal lines represent baseline values (solid line = saline group; dotted line = lidocaine group). n.u. = normalized units.

Except for LF/HF, we found no differences in baseline HRV parameters between the LID and SAL treatments (HR: *P* = 0.74; RMSSD: *P* = 0.74; HF: *P* = 0.11; LF/HF: *P* = 0.01). The AUC values for all parameters in both treatments are summarized in Table 1. We found no significant differences in the AUC parameters of the HRV indices between the LID and SAL treatments.

**Table 1:** Mean (±SEM) area under the curve (AUC) parameters of heart rate variability (HRV) in lactating Holstein-Friesian cows calculated for the first 240 min after receiving epidural injection of lidocaine (n = 8) or saline (n = 8)

Treatment	AUC HRV parameter^1^			
	AUC HR	AUC RMSSD (ms × min)	AUC HF (n.u. × min)	AUC LF/HF (min)
Lidocaine	22,915.1 ± 604.9	1,539.6 ± 142.3	4,906.0 ± 398.2	1,383.1 ± 110.5
Saline	22,534.7 ± 768.0	1,537.2 ± 232.2	4,547.6 ± 512.4	1,561.9 ± 170.9
*P*-value^2^	*P* = 0.84	*P* = 0.98	*P* = 0.31	*P* = 0.19

1 HR = heart rate; RMSSD = the root mean square of successive differences in the consecutive interbeat intervals; HF = the normalized power of the high-frequency band of HRV; LF/HF = the ratio between the low-frequency (LF) and HF components of HRV; n.u. = normalized units.

2 AUC values between lidocaine and saline treatments were compared using the paired *t*-test or by the Wilcoxon signed-rank test.

Analysis of HRV has proven valuable in human anesthesiology research but remains underexplored in veterinary medicine, particularly for cattle (Pagliosa et al., 2015). Our study addresses this knowledge gap by measuring HRV parameters during epidural anesthesia in dairy cows, which previous bovine studies have not specifically investigated.

Our experimental design using saline controls in a crossover study allowed us to differentiate between procedural effects (handling, injection pressure) and pharmacological effects of lidocaine itself. Our findings revealed no differences in AUC values for any HRV parameters between LID and SAL treatments. The cardiovascular stability likely results from the limited cranial spread of lidocaine from the sacrococcygeal junction, which affects only sacral and coccygeal segments while preserving thoracic sympathetic outflow to the heart (Lee and Yamada, 2005).

The stability of RMSSD and HF between treatments further supports our conclusion that epidural lidocaine at the administered dose (4 mL of 2% solution) did not detectably alter vagal modulation. Although systemic lidocaine absorption from the epidural space could theoretically influence HRV parameters, previous studies show undetectable plasma concentrations following caudal epidural administration in cattle (Sellers et al., 2009). Our findings confirm that the dose used (0.15–0.17 mg/kg) does not affect cardiac autonomic regulation as measured by HRV. The baseline difference in LF/HF between treatments likely reflects natural variability in autonomic tone among animals, despite randomization and the crossover design. Postintervention AUC values for LF/HF showed no significant treatment effect, suggesting that epidural lidocaine does not substantially alter sympathovagal balance compared with saline in dairy cows.

Our findings suggest that anatomical differences between injection sites explain why lumbar (Deschamps et al., 2004) and thoracic (Hopf et al., 1990, 1995) approaches affect cardiac sympathetic outflow, whereas our caudal technique does not. Several complementary physiological mechanisms may explain the negligible impact of epidural lidocaine on HRV parameters in dairy cows. First, the anatomical distribution of the sympathetic nervous system (**SNS**) in ruminants differs from humans. In cattle, the sympathetic chain extends from the first thoracic vertebra (T1) to the fourth or fifth lumbar vertebra (L4 or L5), with cardiac sympathetic innervation primarily originating from the cervical and cranial thoracic segments (Sisson and Grossman, 1975). The sacrococcygeal epidural injection employed in our study would not be expected to reach these fibers, thus preserving SNS modulation of cardiac function. Second, the volume and concentration of lidocaine used likely resulted in limited cranial spread within the epidural space. Previous studies have demonstrated that the cranial spread of epidural anesthetics in cattle correlates with the volume administered, with 4 mL typically affecting only the sacral and coccygeal segments (Al-lethie et al., 2023). This localized effect minimizes impact on thoracolumbar sympathetic outflow, preserving cardiovascular autonomic regulation and providing adequate analgesia for caudal procedures. Third, compensatory mechanisms likely maintained autonomic balance despite any partial sympathetic blockade. Baroreceptor reflexes and hormonal responses can rapidly adjust to maintain cardiovascular homeostasis following partial autonomic blockade (Hopf et al., 1990).

Our study has certain limitations that should be acknowledged. First, although the sample size was adequate to detect meaningful physiological differences, subtle effects might have gone undetected. The absence of significant differences does not definitively exclude the possibility of smaller but potentially biologically relevant effects of epidural anesthesia on cardiac autonomic function. Second, measurements were conducted in healthy animals under controlled conditions, and results may differ in stressed or diseased animals. Third, although we used a comprehensive set of HRV parameters, additional measures such as baroreceptor sensitivity might have provided further insights into autonomic regulation. Despite these limitations, our findings validate the use of HRV analysis in dairy cattle welfare studies involving epidural anesthesia. The absence of detectable differences in HRV between LID and SAL treatments suggests that sacrococcygeal epidural anesthesia did not significantly affect cardiac autonomic function in dairy cows under these experimental conditions. From an animal welfare perspective, this has important practical implications for researchers using HRV as a welfare assessment tool, as it confirms that the anesthetic intervention itself does not confound ANS measurements. This enables researchers to confidently attribute observed HRV alterations to procedural stress rather than anesthetic effects when evaluating welfare during common management practices in dairy cattle such as reproductive interventions, minor surgeries, or obstetrical procedures. Furthermore, these results support the use of epidural anesthesia in welfare research protocols without compromising the validity of ANS measurements.

It is important to note that our findings specifically apply to low-dose caudal epidural anesthesia, where motor function remains intact and anesthetic effects are limited to the caudal segments. High-dose epidural techniques that affect more cranial nerve segments may have different effects on cardiac autonomic regulation. Future research should explore dose-dependent effects, agent-specific responses, and the influence of pathological conditions on HRV fluctuations following epidural anesthesia. Such investigations will further refine our understanding of ANS responses to regional anesthesia and inform evidence-based anesthetic protocols for veterinary patients.
